# Association between sarcopenia and new-onset chronic kidney disease among middle-aged and elder adults: findings from the China Health and Retirement Longitudinal Study

**DOI:** 10.1186/s12877-024-04691-1

**Published:** 2024-02-06

**Authors:** Tong Liu, Yang Wu, Xirong Cao, Kun Yang, Yingmu Tong, Fengping Zhang, Cong Wang, Ruixia Cui, Jie Ren, Qinglin Li, Hai Wang, Chang Liu, Jingyao Zhang

**Affiliations:** 1 Department of SICU, The First Affiliated Hospital of Xi’an Jiaotong University, Xi’an, China; 2 Key Laboratory of Surgical Critical Care and Life Support (Xi’an Jiaotong University), Ministry of Education, Xi’an, China; 3 Department of Hepatobiliary Surgery, The First Affiliated Hospital of Xi’an Jiaotong University, Xi’an, China; 4 Department of General Surgery, The First Affiliated Hospital of Xi’an Jiaotong University, Xi’an, China

**Keywords:** Sarcopenia, Chronic kidney disease, Middle-aged and elder adults

## Abstract

**Background:**

Sarcopenia is a senile syndrome of age-related muscle loss. It is thought to affect the development of chronic kidney disease and has a serious impact on the quality of life of the elder adults. Little is known about the association between sarcopenia and new-onset chronic kidney disease in middle-aged and elder adults. Using nationally representative data from the China Health and Retirement Longitudinal Study (CHARLS), we conducted a longitudinal analysis to investigate the association between sarcopenia status and new-onset chronic kidney disease in middle-aged and elder adults in China.

**Methods:**

The study population consisted of 3676 participants aged 45 or older selected from 2011 CHARLS database who had no history of chronic kidney disease at the baseline and completed the follow-up in 2015. A multivariate cox regression model was employed to examine the association between sarcopenia and the incidence of new-onset chronic kidney disease.

**Results:**

Followed up for 4 years, a total of 873 (22.5%) new cases of chronic kidney disease occurred. Among them, participants diagnosed with sarcopenia (HR1.45; 95% CI 1.15–1.83) were more likely to develop new-onset chronic kidney disease than those without sarcopenia. Similarly, patients with sarcopenia were more likely to develop new-onset chronic kidney disease than those with possible sarcopenia (HR 1.27; 95%CI 1.00-1.60). Subgroup analysis revealed that elder adults aged between 60 and 75 years old (HR 1.666; 95%CI 1.20-22.28), with hypertension (HR 1.57; 95%CI 1.02–2.40), people with sarcopenia had a significantly higher risk of developing new-onset chronic kidney disease than those without sarcopenia (all *P* < 0.05).

**Conclusion:**

Middle-aged and elder adults diagnosed with sarcopenia have a higher risk of developing new-onset chronic kidney disease.

**Supplementary Information:**

The online version contains supplementary material available at 10.1186/s12877-024-04691-1.

## Introduction

Sarcopenia is a geriatric syndrome characterized by age-related decreased muscle mass, decreased muscle strength, and(or) decreased physical performance. It is currently estimated that there are 50 million people worldwide with sarcopenia, and this number is expected to reach 500 million by 2050 [1]. As early as 2016, sarcopenia was recognized as one of the most common diseases of the old adults [2]. At present, various international research organizations, such as the European Working Group on Sarcopenia in Older Adults (EWGSOP), Asian Working Group for Sarcopenia (AWGS), International Sarcopenia Working Group (IWGS), and the Foundation for the National Institutes of Health (FNIH) Sarcopenia Project, have developed their own diagnostic criteria for sarcopenia [3–5]. Therefore, the prevalence of sarcopenia varies depending on the different diagnostic criteria and screening methods used. The overall prevalence of sarcopenia in Asian is from 7.8–35.3% [6–8]. In elder adults, sarcopenia is associated with worse outcomes, including physical disability, falls, fractures, poor quality of life, hospitalization and mortality n [9–11]. Moreover, multiple meta-analyses have shown that sarcopenia is associated with all-cause mortality in senile patients (community, nursing home, hospital) [12, 13]. Therefore, sarcopenia diminishes personal quality of life and incurs a heavy medical burden on society. The aging population in our country has made sarcopenia a major concern for medical and social sectors.

Chronic kidney disease (CKD) is one of the major non-communicable chronic diseases in the world, with insidious onset and asymptomatic early stage. Some patients have already entered into end-stage renal disease (ESRD) at the time of treatment. Therefore, early screening and diagnosis are beneficial to timely diagnosis and treatment of the disease, delay the progression, and reduce the incidence of ESRD. Sarcopenia is a common complication of CKD, especially in ESRD patients. Epidemiological data show that the prevalence of sarcopenia in CKD patients is significantly higher than that in the general population [14]. The prevalence of sarcopenia is 5–13% in people aged 60–70 years, and 11–50% in people aged 80 years or older [15]. Depending on the different kinds of diagnostic criteria used for sarcopenia [16, 17], dialysis patients with ESRD have a higher prevalence of sarcopenia, ranging from 25.9 to 34.6%. The presence of characteristics of sarcopenia in patients at all stages of CKD indicates an adverse prognosis [17]. It is possible that the decrease in renal function in CKD impacts patients’ physiological skeletal muscle usage, and its inherent metabolic disease increases protein catabolism. This leads to decreased skeletal muscle mass and weakened functionality [18]. In recent years, it is also becoming clear that there is an important crosstalk between kidney and muscle. Pathologic occurrences in the kidney, such as metabolic acidosis and increased inflammatory factors, insulin and alterations in microRNAs, may negatively impact protein metabolism in skeletal muscle, which is also capable of secreting various signaling molecules, such as myokines, bioactive peptides, lipids and microRNAs, that communicate with receptor organs, including the kidney [19, 20]. It has been found that metabolic markers of sarcopenia overlap with uremic compounds that are increased in renal insufficiency or uremia, providing a potential theoretical basis for the occurrence of nephropathy in patients with sarcopenia [21].

In summary, recent research on sarcopenia and kidney disease has primarily focused on the prevalence of sarcopenia in patients with CKD and the adverse effects of combined sarcopenia on their quality of life and mortality, while the incidence of kidney disease in patients with sarcopenia has not been reported. In other words, the effect of individual sarcopenia on kidney disease remains uncertain. Therefore, using nationally representative data from the China Health and Retirement Longitudinal Study (CHARLS), we conducted a longitudinal analysis to investigate the association between sarcopenia status and new-onset chronic kidney disease among middle-aged and elder adults in China.

## Method

### Study population

**Fig. 1:**
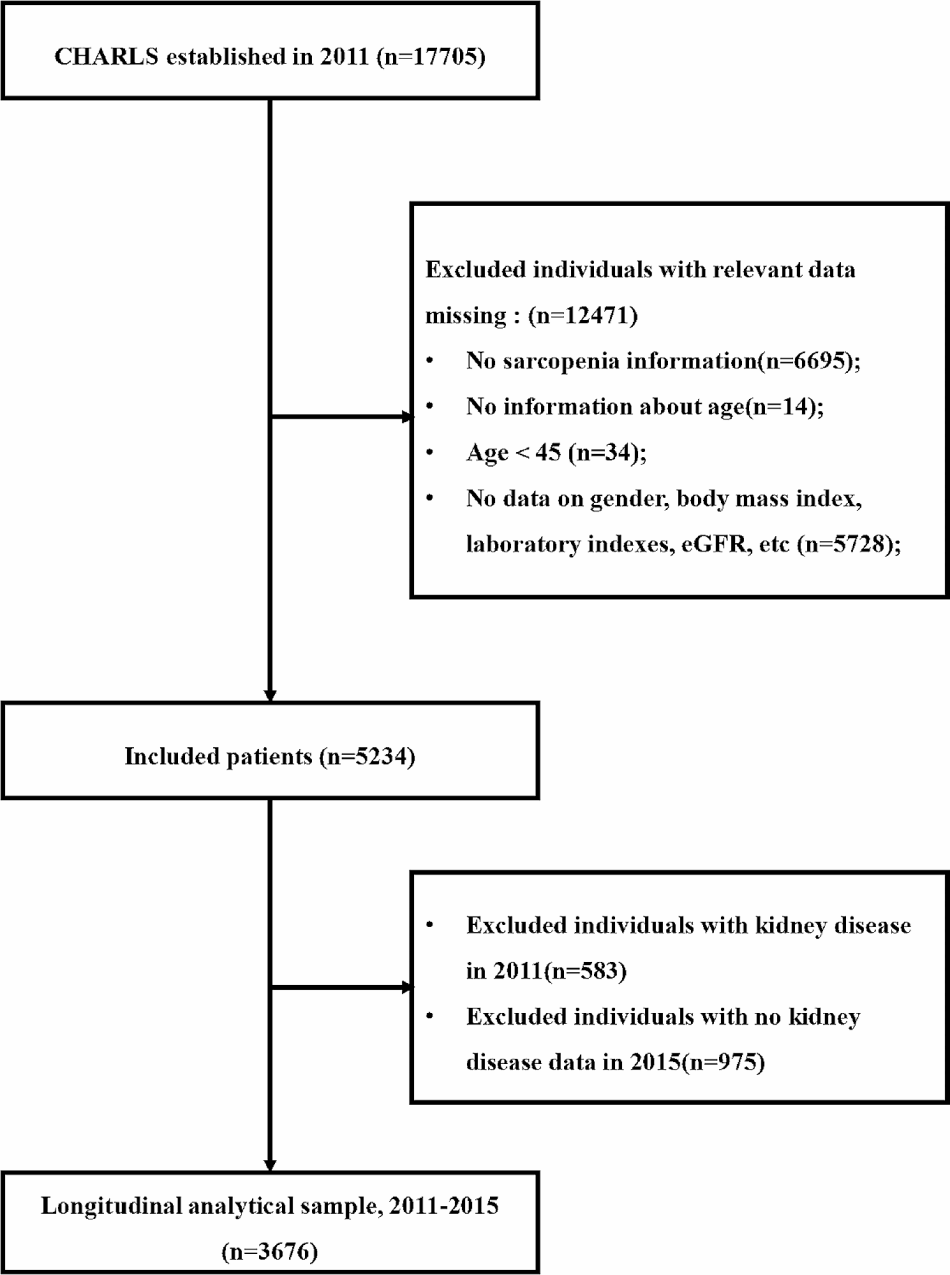
Flow diagram for participants included in the study

The CHARLS, established in 2011, is a nationally representative longitudinal survey in China, which collects high-quality data from a nationally representative sample of Chinese population through one-on-one interviews and structured questionnaires [22]. In this study, we retrospectively analyzed CHARLS data from 2011 to 2015. The inclusion criteria for this study were: 1) individuals aged at least 45 years old in CHARLS 2011 and had data on sarcopenia status. Exclusion criteria were: 1) missing data on sarcopenia status in CHARLS 2011 no age information; (3) age < 45 years old; (4) Lack of gender, body mass index and laboratory data, estimated glomerular filtration rate (eGFR), etc. In the first step of population screening, we used data from the large follow-up cohort of the CHARLS 2011. A total of 17,705 participants were surveyed in CHARLS 2011, 12,471 were excluded due to missing data on sarcopenia (*n* = 6695), no age information (*n* = 14), age less than 45 years (*n* = 34), lack of data on body mass index and blood pressure (*n* = 5728). Remaining 5234 participants We also excluded 583 individuals with kidney disease in CHARLS 2011 and 975 individuals with lacking kidney disease data in CHARLS 2015. Finally, our sample consisted of 3676 individuals who were no kidney disease in CHARLS 2011 and had a full follow-up in 2015. The selection procedure is shown in detail in Fig. 1.

All participants provided written informed consent, which was approved by the Biomedical Ethics Review Committee of Peking University. All procedures in this study involving human participants were performed in accordance with the ethical standards of the institutional and/or National Research Council and the 1964 Declaration of Helsinki and its subsequent amendments or similar ethical standards. The study was conducted according to the Reporting of Enhanced Observational Studies in Epidemiology (STROBE) guidelines [23].

### Assessment of the sarcopenia state and chronic kidney disease

Sarcopenia status is assessed according to the AWGS 2019 algorithm, which has three components: muscle strength, muscle mass, and physical performance. AWGS 2019 recommends the use of handgrip strength to represent muscle strength [24]. Handgrip strength (kg) is measured using both dominant and non-dominant hands. The patient is asked to test grip strength twice on each hand and the maximum grip strength is used for analysis. Low muscle strength in men and women is defined as grip strength < 28 kg and < 18 kg, respectively. Muscle mass is estimated from appendiceal skeletal muscle mass (ASM), which has been previously formulated [25], and previous studies have shown good agreement between ASM equation model and dual X-ray absorptiometry (DXA) [25, 26]. The cut-off point for low muscle mass is based on height-adjusted muscle mass (ASM/Ht^2^) in the lowest 20% of the study population [27–29]. In our study, the ASM/Ht^2^ values < 8.79 kg/m^2^ in women and ASM/Ht^2^ value < 6.84 kg/m^2^ in men were considered low muscle mass. For physical performance, gait speed and chair standing test methods are used for assessment, to be specific, the criteria for low physical performance are 6-m walk < 1.0 m/s, or 5-time chair stand test ≥ 12 s. No sarcopenia was defined as normal for all three aspects. Possible sarcopenia was defined by low muscle strength with or without reduced physical performance. Sarcopenia was diagnosed when low muscle mass plus low muscle strength or low physical performance are detected [24]. Further details regarding the definition of the sarcopenia component in CHARLS had been described previously [27].

Chronic kidney disease was diagnosed according to one of the following criteria: (1) Self-report of physician diagnoses; (2) eGFR < 60 ml/min/1.73m^2^. The eGFR were assessed using the Chronic Kidney Disease-Epidemiology Collaboration (CKD-EPI) formula, which included serum creatinine values, age, sex and race to estimate GFR [30].

### Covariates

Previous research indicates that the influences linked with kidney disease and sarcopenia include not only self-inflicted factors like gender, age, and comorbidities, but also the socioeconomic factors and standard of living [4, 24, 31, 32]. Additionally, meta-analysis provides evidence that cigarette smoking is an independent risk factor for developing CKD [33] and sarcopenia [34]. In contrast to smoking, the effects of alcohol on the kidneys remain a topic of controversy within the scientific community [35, 36]. Studies indicate that smoking and chronic excessive drinking can be reduced through various pathways that impact energy intake and increase muscle growth inhibition and muscle catabolism [37]. Therefore, our study collected information on sociodemographic status and health-related factors. Sociodemographic variables included age, gender, educational attainment (primary school or below, secondary school, university or higher), marital status (married or other), and residential location (rural or urban). Health-related factors encompassed body mass index (BMI), hypertension, diabetes, and dyslipidemia, the history of smoking and drinking (yes or no). In particular, these diseases are diagnosed according to the following criteria.

Hypertension was diagnosed when systolic blood pressure (SBP) was ≥ 140 mmHg, diastolic blood pressure (DBP) was ≥ 90mmHg, or use of antihypertensive medication in the self-report, or when a physician diagnosed it in the self-report.

Diabetes was diagnosed according to one of the following criteria: (1) self-reported physician diagnosis; (2) glycosylated hemoglobin (HbA1c) ≥ 6.5%; (3) blood glucose ≥ 126 mg/dL (fasting), or blood glucose ≥ 200 mg/dL (casual); (4) self-reported hypoglycemic or insulin treatment.

Dyslipidemia was based on one of the following criteria:(1) self-reported physician diagnosis; Total cholesterol (TC) ≥ 240 mg/dL; High density lipoprotein cholesterol (HDL) ≤ 40 mg/dL; 4) low-density lipoprotein cholesterol (LDL) ≥ 160 mg/dL; (5) triglyceride (TG) ≥ 150 mg/dL; (6) self-description of anti-dyslipidemia treatment.

Smoking was assessed by asking the following question: “Have you ever chewed tobacco, smoked a pipe, smoked self-rolled cigarettes, or smoked cigarettes or cigars?” Those who responded “no” were considered non-smokers, while those who responded “yes” were labelled as smokers. Drinking was evaluated through the question: “Have you consumed any alcoholic beverages, such as beer, wine, liquor or rice wine per month in the last year?” During the interview, participants were asked about their history of alcohol drinking using the following options: (1) I never had a drink; (2) I used to drink less than once a month; (3) I used to drink more than once a month. In our study, option 2 and option 3 were considered indicative of a history of alcohol drinking.

### Statistical analysis

All statistical analysis were performed retrospectively with 26.0 (SPSS, Inc., Chicago, IL). Continuous variables were represented by mean and standard deviation (SD) for normal distribution and median and quartile difference for skewed distribution. One-way ANOVA was used to compare differences between the three groups (non-sarcopenia, possible sarcopenia and sarcopenia). Categorical variables were expressed as counts and percentages, and chi-square test was used for comparison between groups. We also measured follow-up time, which was defined as the time from the date of the first interview after enrollment to the end point of the study. The endpoint of the study was the date of new-onset chronic kidney disease diagnosis or the date of the subsequent interview (July 2015). To estimate the association between baseline sarcopenia status and incident kidney disease, Cox proportional-hazards models were used to calculate hazard ratios (HRs) and 95% confidence intervals (CIs), and their interactions were tested. The person-year incidence rate was calculated by dividing the number of cases in this population during the period of follow-up by the person-years of follow-up. The person-year was the sum of the duration of follow-up without disease for all persons followed. *P* < 0.05 was considered to be statistically significant.

## Results

### Characteristics of baseline population

A total of 3676 participants were included in our study. Table 1 showed the characteristics of participants with different sarcopenia states. The median age of all participants was 63 years old (interquartile range, 60–69 years old), with 1691 males (46%). The prevalence of sarcopenia and possible sarcopenia was 17.7% (649/3676) and 52.8% (1941/3676), respectively. Compared with those without sarcopenia, participants with sarcopenia were older (median age, 61.0 vs. 68.0 years old), had fewer males (77.7% vs. 88.8%), resided more in rural areas (78.4% vs. 65.7%), had a lower prevalence of hypertension (45.3% vs. 60.8%) and dyslipidemia (31.0% vs. 51.9%), and lower BMI (18.71vs23.77) (all *P* < 0.001).

**Table 1:** Characteristics of all participants by sarcopenia status

Characteristics	Overall (*n* = 3676)	No sarcopenia (*n* = 1086)	Possible sarcopenia (*n* = 1941)	Sarcopenia (*n* = 649)	*P*
Age, year	63.00 (60.00, 69.00)	64.00 (62.00, 68.00)	61.00 (55.00, 68.00)	68.00(62.00, 74.00)	< 0.001
Male, *n* (%)	1691 (46.0)	631 (58.1)	764 (39.4)	296 (45.6)	< 0.001
Married (vs others)	3128 (85.1)	964 (88.8)	1660 (85.5)	504 (77.7)	< 0.001
Rural (vs urban)	2559 (69.6)	714 (65.7)	1336 (68.8)	509 (78.4)	< 0.001
Smoking	1500 (40.8)	437 (40.3)	796 (41.4)	267 (41.1)	0.893
Drinking	1160 (31.6)	341 (31.4)	601 (31.0)	218 (33.6)	0.477
**Educational level**					< 0.001
Elementary school or below	2949 (80.2)	844 (77.7)	1549 (79.8)	556 (85.8)	
Secondary school	689 (18.7)	222 (20.4)	378 (19.5)	89 (13.7)	
College and above	36 (1.0)	20 (1.8)	13 (0.7)	3 (0.5)	
**Comorbidities**, ***n*(%)**					
Hypertension	2108 (57.3)	658 (60.8)	1158 (59.8)	292 (45.3)	< 0.001
Dyslipidemia	1775 (48.3)	559 (51.9)	1017 (52.8)	199 (31.0)	< 0.001
Diabetes	735 (20.3)	243 (22.6)	401 (20.8)	91 (14.1)	0.323
**Blood pressure, mm Hg**					
Systolic	130.00(116.33, 145.33)	131.33 (118.67, 146.33)	130.33 (116.50, 146.33)	126.33 (113.00, 140.67)	< 0.001
Diastolic	74.33 (66.67, 82.33)	74.83 (67.67, 82.33)	75.33 (67.67, 83.67)	70.67 (63.00, 78.33)	< 0.001
**Laboratory test indicators**					
Glucose (mg/dl)	102.60 (95.04, 114.02)	103.68 (96.12, 117.36)	103.14 (95.22, 114.30)	100.26 (92.88, 109.26)	< 0.001
Creatinine (mg/dl)	0.76 (0.66, 0.87)	0.79 (0.69, 0.93)	0.73 (0.63, 0.85)	0.75 (0.64, 0.85)	< 0.001
Total cholesterol, mg/dL	191.37 (167.78, 216.11)	193.30 (168.17, 217.27)	190.98 (168.56, 216.88)	189.05 (165.08, 212.05)	0.040
Triglycerides, mg/dL	103.55 (74.34, 149.57)	104.43 (75.23, 155.76)	109.74 (78.77, 160.19)	84.96 (65.49, 120.36)	< 0.001
LDL cholesterol, mg/dL	115.59 (93.94, 138.40)	118.68 (95.88, 140.72)	48.33 (39.43, 58.38)	56.44 (47.17, 68.04)	< 0.001
HDL cholesterol, mg/dL	49.48 (40.59, 59.92)	47.55 (39.82, 58.76)	115.59 (94.72, 139.18)	112.89 (89.11, 132.02)	< 0.001
Height, cm	156.70 (151.00, 163.00)	158.50 (152.00, 164.73)	155.90 (150.55, 162.30)	156.00 (150.30, 161.00)	< 0.001
Weight, kg	56.20 (49.50, 64.40)	53.00 (59.10, 66.40)	58.60 (52.70, 65.80)	45.50 (41.45, 48.80)	< 0.001
eGFR, mL/min/1.73 m^2^	92.57 (83.06, 99.22)	90.60 (81.40, 96.42)	94.43 (84.74, 101.15)	90.41 (80.53, 97.24)	< 0.001
**Handgrip strength, kg**					
Male	36.20(31.00,42.00)	39.00(34.60,44.00)	35.50(29.00,41.98)	32.40(27.00,36.50)	< 0.001
Female	25.00(20.00,29.60)	26.10(22.50,30.00)	25.00(20.00,30.00)	23.00(19.00,27.50)	< 0.001
**ASM/Ht**^**2**^, **kg/m**^**2**^					
Male	7.39(6.99,7.90)	7.57(7.18,8.04)	7.54(7.19,8.05)	6.63(6.42,6.75)	< 0.001
Female	9.44(8.96,9.94)	9.56(9.19,9.91)	9.63(9.22,10.13)	8.48(8.27,8.66)	< 0.001
Gait speed, m/s	1.27 (1.00, 1.54)	1.46 (1.25, 1.70)	1.03 (0.82, 1.34)	1.23 (0.95, 1.49)	< 0.001
5-time chair stand test, s	12.03 (9.34, 14.52)	9.22 (7.81, 10.44)	13.50 (12.22, 15.62)	11.56 (9.31, 14.02)	< 0.001

Abbreviation: ASM, appendicular skeletal muscle; BMI, body mass index; LDL, low-density lipoprotein; HDL, high-density lipoprotein; eGFR, estimated glomerular filtration rate.

### The risk factors of new-onset chronic kidney disease

Firstly, we established a univariate Cox regression model to identify potential risk factors for new-onset kidney disease, and the results are displayed in Table 2. We found that age (HR 1.02; 95%CI 1.02–1.03), sarcopenia (HR 1.48; 95%CI 1.23–1.79), HDL (HR 1.01; 95%CI 1.00-1.01) and drinking (HR 1.19; 95%CI 1.04–1.37) were risk factors for new-onset chronic kidney disease while height, weight, and eGFR were protective factors for new-onset chronic kidney disease, but the difference of HR value was not obvious (all *P* < 0.05).

**Table 2:** Univariate cox hazard analyses of risk factors for new-onset kidney disease

**Variables**	**HR (95%CI)**	***P***
Age	1.02 (1.02–1.03)	< 0.001
Sarcopenia status		
No sarcopenia	1.00 (Ref.)	
Possible sarcopenia	1.06 (0.91–1.25)	0.430
Sarcopenia	1.48 (1.23–1.79)	< 0.001
Drinking	1.19(1.04–1.37)	0.013
Handgrip strength, kg	0.99 (0.98–0.99)	< 0.001
Gait speed, m/s	0.83 (0.70–0.99)	0.040
Height, cm	0.99 (0.98–0.99)	0.001
Weight, kg	0.99 (0.98-1.00)	< 0.001
eGFR, mL/min/1.73 m^2^	0.99 (0.99-1.00)	0.003
HDL cholesterol, mg/dL	1.01 (1.00-1.01)	0.032

Abbreviation: HRs, hazard ratios; CI, confidential interval; HDL, high-density lipoprotein; eGFR, estimated glomerular filtration rate.

### Association between sarcopenia and new-onset chronic kidney disease

A total of 875 (23.8%) participants developed new-onset chronic kidney disease events during the 4 years of follow-up. Specifically, the incidence of new-onset chronic kidney disease was 59.38/1000 person-years in patients without sarcopenia, 62.93/1000 person-years in those with possible sarcopenia, and 86.98/1000 person-years in subjects with sarcopenia. Table 3 also showed the association between baseline sarcopenia status and new-onset chronic kidney disease. Models 2–4 illustrated that, after adjusting for covariates, compared with those without sarcopenia, individuals with possible sarcopenia (HR 1.12; 95%CI 0.95–1.32) and participants with sarcopenia were more likely to develop new-onset chronic kidney disease (HR 1.45; 95%CI 1.15–1.83), which means sarcopenia was a risk factor for new-onset kidney disease (*P* < 0.01). According to Table S1, the proportion of patients with sarcopenia who had all stages of CKD was slightly higher compared to those without sarcopenia. Similarly, individuals with sarcopenia were more likely to develop new-onset chronic kidney disease than those with possible sarcopenia (HR 1.27; 95%CI 1.00-1.60; *P* < 0.05) (table S2). To test the stability of the conclusions, participants with eGFR of 90 or more (*N* = 2133) were selected for sensitivity analysis, and the results were shown in Table 4. Consistently, people with sarcopenia were more likely to develop new-onset chronic kidney disease events than those without sarcopenia. (HR 1.6; 95%CI 1.19–2.2; *P* < 0.05).

**Table 3:** Incidence of new-onset kidney disease according to baseline sarcopenia status, 2011?2015

	Cases (*n*)	Incidence Rate, per 1000 Person-Years	HR (95%CI)				
			Model 1	Model 2	Model 3	Model 4	
No sarcopenia	235	59.38	1.00 (Ref.)	1.00 (Ref.)	1.00 (Ref.)	1.00 (Ref.)	
Possible sarcopenia	439	62.93	1.06 (0.91–1.25)	1.12 (0.96–1.32)	1.12 (0.95–1.32)	1.12 (0.95–1.32)	
Sarcopenia	201	86.98	1.48 (1.23–1.79)***	1.36 (1.12–1.65)**	1.47 (1.17–1.86)**	1.45 (1.15–1.83)**	

• Abbreviation: HRs, hazard ratios; CI, confidential interval; BMI, body mass index; SBP, systolic blood pressure; DBP, diastolic blood pressure, eGFR, estimated glomerular filtration rate.

• Model 1 was unadjusted

• Model 2 was adjusted for age, sex

• Model 3 was adjusted for age, sex, drinking, height, weight, eGFR, HDL cholesterol

• Model 4 was adjusted for age, sex, residence, marital status, educational level, smoking, drinking, BMI, SBP, DBP; history of hypertension, dyslipidemia, diabetes

• Significant at * *P* < 0.05** *P* < 0.01.*** *P* < 0.001

**Table 4:** Sensitivity analysis of association between sarcopenia and new-onset kidney diseases in CHARLS (2011–2015) excluding individuals with eGFR less than 90

	Cases (n)	Incidence Rate, per 1000 Person-Years	HR (95%CI)			
			Model 1	Model 2	Model 3	Model 4
No sarcopenia	116	54.84	1.00 (Ref.)	1.00 (Ref.)	1.00 (Ref.)	1.00 (Ref.)
Possible sarcopenia	258	55.97	1.04 (0.84–1.29)	1.14 (0.91–1.43)	1.14 (0.90–1.43)	1.13 (0.90–1.42)
Sarcopenia	111	91.40	1.67 (1.28–2.17)***	1.60 (1.23–2.08)***	1.51 (1.15–1.97)**	1.64 (1.19–2.26)**

• Abbreviation: HRs, hazard ratios; CI, confidential interval; BMI, body mass index; SBP, systolic blood pressure; DBP, diastolic blood pressure.

• Model 1 was unadjusted

• Model 2 was adjusted for age, sex

• Model 3 was adjusted for age, sex, drinking, height, weight, eGFR, HDL cholesterol

• Model 4 was adjusted for age, sex, residence, marital status, educational level, smoking, drinking, BMI, SBP, DBP; history of hypertension, dyslipidemia, diabetes, stroke, heart disease

• Significant at * *P* < 0.05** *P* < 0.01.*** *P* < 0.001

### Subgroup analysis of relationship between sarcopenia status (No sarcopenia and Sarcopenia) and new-onset chronic kidney disease

Based on the findings of this research, we identified two cohorts of individuals, one with sarcopenia and the other without, to investigate the correlation between sarcopenia and the new-onset chronic kidney disease. Sex, age, hypertension, diabetes, drinking, and smoking were grouped measures for our subgroup analysis, and adjusted covariables including age, sex, residence, marital status, educational level, smoking, drinking, BMI, SBP, DBP; history of hypertension, dyslipidemia, diabetes. The results are presented in Table S3. We found that individuals with sarcopenia aged between 60 and 75 years old (HR 1.66; 95%CI 1.20-22.28), with hypertension (HR 1.57; 95%CI 1.02–2.40), were significantly more likely to develop new-onset chronic kidney disease than those without sarcopenia (all *P* < 0.05).

## Discussion

Sarcopenia is a condition characterized by decreased skeletal muscle mass, strength and physical function. According to the etiology, it can be categorized into age-related primary sarcopenia and secondary sarcopenia, with the latter exhibiting more serious and dramatic muscle mass loss due to the presence of protein degradation factors. Chronic kidney disease can lead to increased skeletal muscle catabolism, which is an important factor promoting the occurrence of secondary sarcopenia. Previous studies have primarily focused on the risk of developing sarcopenia in CKD patients and the impact of combined sarcopenia on the prognosis of CKD patients. However, no studies have investigated whether sarcopenia increases the risk of developing kidney disease in the old population. In this study, we conducted a longitudinal analysis of 3,676 individuals from the CHARLS 2011 database and found that sarcopenia, as assessed by AWGS 2019 algorithm, was independently and positively associated with new-onset chronic kidney disease. What’s more, in middle-aged and elder adults, patients diagnosed with sarcopenia had a higher risk of new-onset chronic kidney disease, and there was no clear difference in the risk of new kidney disease between patients with possible sarcopenia and individuals without sarcopenia.

Our study identified several risk factors for new-onset chronic kidney disease, including age, HDL, and alcohol consumption, which was consistent with previous studies [38–41]. In model 3, we corrected for statistically significant factors for new-onset chronic kidney disease based on one-way cox regression analyses and excluded variables involved in the assessment of sarcopenia. The results showed that sarcopenia was an independent risk factor for new-onset chronic kidney disease. In addition, sensitivity analysis was performed for further verification, and the consistent results increased the credibility of our conclusions. Our results showed that patients with sarcopenia had a significantly higher risk of new-onset chronic kidney disease, both compared with those without sarcopenia and those with possible sarcopenia. Our subgroup analysis indicated that non-diabetics with sarcopenia had a significantly higher risk of developing new-onset chronic kidney disease than those without sarcopenia. Consistent with our results, Han [42] also found that pre-sarcopenia increased renal hyperfiltration risk in the non-diabetic subgroup after adjusting for confounding factors. This study also suggested that sarcopenia may had adverse effects on kidney function. In the diabetic group, patients with sarcopenia continued to show an increased risk of new-onset chronic kidney disease, but the results were not statistically significant. Previous studies have reported similar rates of kidney disease in type 2 diabetes mellitus (T2DM) patients with or without muscle loss [43, 44]. Previous studies have shown that hypertension was an independent risk factor for chronic kidney disease caused by various types of kidney disease [45, 46], but Gao [47] found that hypertension was not associated with an increased risk of sarcopenia, which was consistent with our results in the univariate regression analysis. Additionally, our findings reveal no correlation between sarcopenia and hypertension. Patients diagnosed with both conditions, however, exhibit a greater risk of developing new-onset chronic kidney disease. This suggests that hypertension and sarcopenia may play separate roles in the development of kidney damage. Nevertheless, further epidemiological research is needed to fully understand the role of hypertension in the development of kidney damage in people with sarcopenia.

Sarcopenia is a complex disease caused by multiple factors, including aging, endocrine alterations, chronic diseases, inflammation, mitochondrial dysfunction, lack of exercise, cachexia, genetic factors, etc. This condition leads to the degeneration and atrophy of skeletal muscle in the body. Third National Health and Nutrition Examination Survey (NHANES III) [48] previously reported an increased prevalence of sarcopenia in individuals with decreased kidney function. However, the mechanism by which sarcopenia impinges on kidney function remains unclear. It has been shown that the signature metabolites in the blood of patients with sarcopenia include the tricarboxylic acid (TCA) cycle, urea cycle, nitrogen and methylated metabolites, which are uremic compounds with impaired renal dysfunction, suggesting a close link between muscle and renal function [21]. We propose that sarcopenia results in a significant metabolic waste burden. The majority of these metabolites are excreted by the kidneys. However, due to the excessive amounts of metabolites and the increased of stress on the kidney, it may not be able to effectively remove waste from the body, leading to kidney damage and other health problems. Moreover, sarcopenia is an independent risk factor for albuminuria, and this association is particularly strong in elder adults [49]. Albuminuria can accelerate tubular injury through various ways [50], and it is a predictor of the CKD progression [51]. In addition, the expression of vitamin D receptor in skeletal muscle decreases due to aging, and the functional response to vitamin D reduces, leading to decreased muscle mass and strength [52–54]. A recent study by Girgis [55] has demonstrated that the presence of vitamin D receptor (VDR) in myosphere in mice, and the deletion of VDR is associated with lean body mass loss, muscle loss, and decreased grip strength and motor performance. At the same time, patients with sarcopenia have limited activities, which further aggravates vitamin D deficiency. This deficiency can cause damage to active vitamin D’s protection of renal cystopodia cells, increased albuminuria excretion, renal inflammatory response and fibrosis, and ultimately increased renal function impairment [56, 57].

However, our study still has some limitations. First of all, observational data is used in this study, and the observational relationship may be biased due to confounding factors. To reduce this bias, we considered as many relevant factors as possible in our analysis. However, other potential confounding factors, such as body fat mass and lack of exercise, cannot be ruled out. Second, there are no medical records in CHARLS, so there may also be some degree of bias in the use of self-reported measures of chronic disease. We further checked the diagnosis using specific test measures to reduce the degree of bias. Third, due to the lack of a detailed classification of kidney disease, we were unable to conduct an in-depth analysis of the relationship between sarcopenia and specific kidney disease. Finally, when explaining and inferring our results, some types of choice bias, such as potential volunteer deviations and non-reactive deviations, should also be considered. Despite these limitations, this study helps to expand our previous understanding of the impact of muscular disease on CKD. It is recommended that assessments of muscle mass be incorporated into community health check-ups and routine clinical practice. The focus should be on monitoring changes in diagnostically relevant indicators of renal function in older adults who suffer from muscle weakness. This approach aims to decrease the incidence of CKD. Finally, exploring the effect of sarcopenia on the different stages of CKD and looking for its influence on the progression of chronic kidney disease is also a very valuable question. Future studies need to elucidate these important questions.

## Conclusion

This study shows that the incidence of new-onset chronic kidney disease has continued to rise in middle-aged and elder adults with the aggravation of sarcopenia. The patients with sarcopenia have a higher risk of new-onset chronic kidney disease, especially in high blood pressure people. However, there are no clear differences in the risk of new-onset chronic kidney disease between individuals with possible sarcopenia and no sarcopenia.

### Electronic supplementary material

Below is the link to the electronic supplementary material.

Supplementary Material 1

## Data Availability

The data involved in this study are publicly available and can be found at: https://charls.pku.edu.cn/.

## Abbreviations

CHARLS: China Health and Retirement Longitudinal Study
EWGSOP: Working Group on Sarcopenia in Older Adults
AWGS: Asian Working Group for Sarcopenia
IWGS: International Sarcopenia Working Group
FNIH: Foundation for the National Institutes of Health
CKD: Chronic kidney disease
ESRD: End-stage renal disease
STROBE: Enhanced Observational Studies in Epidemiology
ASM: Appendiceal skeletal muscle mass
DXA): Dual X-ray absorptiometry
ASM/Ht^2^: Height-adjusted muscle mass
eGFR: Estimated glomerular filtration rate
BMI: Body mass index
TC: Total cholesterol
HDL: High density lipoprotein cholesterol
LDL: Low-density lipoprotein cholesterol
TG: Triglyceride
HRs: Hazard ratios
CIs: Confidence intervals
SBP: Systolic blood pressure
DBP: Diastolic blood pressure
TCA: Tricarboxylic acid
VDR: Vitamin D receptor
T2DM: Type 2 diabetes mellitu
