# Supplementary material for: Association between sarcopenia and new-onset chronic kidney disease among middle-aged and elder adults: findings from the China Health and Retirement Longitudinal Study
Source: BMC Geriatr. 2024 Feb 6;24:134. doi: 10.1186/s12877-024-04691-1 (PMC10848350; doi:10.1186/s12877-024-04691-1)
Supplement: Supplementary file 1 — Supplementary Material 1 [file 12877_2024_4691_MOESM1_ESM.docx]

Supplementary Material

Association between sarcopenia and new-onset chronic kidney disease among middle-aged and elder adults: Findings from the China health and retirement longitudinal study

# Supplementary Table

| \| Supplementary table1. Proportion of new-onset CKD (by different 5 stages of CKD) in sarcopenia and non-sarcopenia populations \| \| \| \| \| \| \| \| --- \| --- \| --- \| --- \| --- \| --- \| --- \| \|  \| No CKD, n (%) \| different stages of CKD, n (%) \| \| \| \| \| \|  \| Stage-1 \| Stage-2 \| Stage-3 \| Stage-4 \| Stage-5 \| \| No sarcopenia \| 851(78.4%) \| 83(7.6%) \| 56(5.2%) \| 65(6.0%)) \| 10(0.9%) \| 21(1.9%) \| \| Sarcopenia \| 448(69.0%) \| 65(10.0%) \| 58(8.9%) \| 55(8.5%) \| 5(0.8%) \| 18(2.8%) \| \| Abbreviation: CKD, chronic kidney disease. \| \| \| \| \| \| \|   Supplementary table2. Incidence of new-onset kidney disease according to baseline sarcopenia status among Possible sarcopenia and Sarcopenia, from 2011 to 2015 | | | | | | |
| --- | --- | --- | --- | --- | --- | --- | --- | --- | --- | --- | --- | --- | --- | --- | --- | --- | --- | --- | --- | --- | --- | --- | --- | --- | --- | --- | --- | --- | --- | --- | --- | --- | --- | --- | --- | --- | --- | --- | --- | --- | --- | --- | --- | --- | --- | --- | --- |
|  | Cases(n) | Incidence Rate, per 1000 Person-Years | HR(95%CI) | | | |
|  |  |  | Model 1 | Model 2 | Model 3 | Model 4 |
| Possible sarcopenia | 439 | 62.93 | 1.00 (Ref.) | 1.00 (Ref.) | 1.00 (Ref.) | 1.00 (Ref.) |
| Sarcopenia | 201 | 86.98 | 1.39 (1.17-1.64)*** | 1.21 (1.01-1.44)* | 1.29 (1.02-1.64)* | 1.27 (1.00-1.60)* |
| - Abbreviation: HRs, hazard ratios; CI, confidential interval; BMI, body mass index; SBP, systolic blood pressure; DBP, diastolic blood pressure, eGFR, estimated glomerular filtration rate. - Model 1 was unadjusted - Model 2 was adjusted for age, sex - Model 3 was adjusted for age, sex, drinking, height, weight, eGFR, HDL cholesterol - Model 4 was adjusted for age, sex, residence, marital status, educational level, smoking, drinking, BMI, SBP, DBP; history of hypertension, dyslipidemia, diabetes - Significant at * P < 0.05** P< 0.01.*** P <0.001. | | | | | | |

| Supplementary table3. Subgroup analysis of relationship between sarcopenia status (No sarcopenia and Sarcopenia) and new-onset kidney disease. | | | | | |
| --- | --- | --- | --- | --- | --- |
| Characteristics | No sarcopenia | Sarcopenia | HR (95%CI) | P | P for interaction |
| **All patients** | 235/1086 | 200/649 | 1.45 (1.15-1.83) | <0.01 |  |
| **Gender** |  |  |  |  | 0.97 |
| Male | 132/631 | 92/296 | 0.68 (0.45-1.04) | 0.073 |  |
| Female | 103/455 | 108/353 | 0.74 (0.47-21.16) | 0.188 |  |
| **Age** |  |  |  |  | 0.004 |
| ≥ 75 | 12/44 | 43/142 | 1.23 (0.46-3.30) | 0.681 |  |
| ≥60,<75 | 222/1030 | 145/438 | 1.66 (1.20-2.28) | 0.002 |  |
| < 60 | 1/12 | 12/69 | 5.68 (0.04-817.48) | 0.493 |  |
| **Hypertension** |  |  |  |  | 0.778 |
| With | 133/658 | 88/292 | 1.57 (1.02-2.40) | 0.039 |  |
| Without | 102/425 | 110/353 | 1.38 (0.90-2.12) | 0.143 |  |
| **Diabetes** |  |  |  |  | 0.091 |
| With | 59/243 | 33/91 | 1.54 (0.77-3.07) | 0.219 |  |
| Without | 173/834 | 166/555 | 1.40 (0.99-1.97) | 0.055 |  |
| **Drinking** |  |  |  |  | 0.061 |
| With | 85/341 | 77/218 | 1.48 (0.89-2.47) | 0.133 |  |
| Without | 150/744 | 123/431 | 1.41 (0.96-2.07) | 0.077 |  |
| **Smoking** |  |  |  |  | 0.022 |
| with | 99/437 | 93/267 | 1.46 (0.92,2.32) | 0.105 |  |
| without | 136/648 | 107/382 | 1.44 (0.96,2.18) | 0.08 |  |
| - Abbreviation: HRs, hazard ratios; CI, confidential interval; BMI, body mass index; SBP, systolic blood pressure; DBP, diastolic blood pressure. - All models were adjusted by age, sex, residence, marital status, educational level, smoking, drinking, BMI, SBP, DBP; history of hypertension, dyslipidemia, diabetes; unless the variable was used as a subgroup variable | | | | | |
